# Recovery After Ureterorenoscopy and Percutaneous Nephrolithotomy: A Questionnaire Study

**DOI:** 10.3390/jcm15145326

**Published:** 2026-07-08

**Authors:** Angelique M. M. Kleijweg, Riemer A. Kingma, Jinne H. J. Stiksma, Jaap G. H. Poerink, Stijn Roemeling

**Affiliations:** 1 Department of Urology, University of Groningen, University Medical Center Groningen, 9713 GZ Groningen, The Netherlands; 2 Department of Urology, Wilhelmina Hospital Assen, 9401 RK Assen, The Netherlands; 3 Department of Urology, Martini Hospital Groningen, 9728 NT Groningen, The Netherlands

**Keywords:** urolithiasis, kidney stones, ureterorenoscopy (URS), percutaneous nephrolithotomy (PCNL), recovery

## Abstract

**Objectives:** Patient information regarding postoperative recovery after percutaneous nephrolithotomy (PCNL) and retrograde ureterorenoscopy (URS) remains limited. This study aimed to characterize recovery patterns following these procedures to improve patient counseling. **Methods:** In this prospective multicenter study, adult patients undergoing URS or PCNL for renal stones were included at an academic center, a teaching hospital, and a regional hospital. Patients completed questionnaires two weeks postoperatively, assessing demographics and recovery outcomes. If recovery was incomplete, follow-up assessments were performed at five and eight weeks postoperatively. Primary endpoints were time to full recovery, duration of pain, and fatigue. **Results:** Seventy-one patients completed the questionnaire (response rate 56%): 37 underwent PCNL, and 34 underwent URS. The median time to full recovery was 15 days overall: 15 days after PCNL and 13 days after URS. Median postoperative pain duration was 3 days (4 days after PCNL and 2 days after URS). Fatigue was the most frequently reported complaint at two weeks postoperatively, with a median duration of 9 days overall (12 days after PCNL and 7 days after URS). No clear differences in overall recovery duration were observed between URS and PCNL. However, variability in recovery outcomes was considerable. **Conclusions:** This exploratory study provides early prospective insight into patient-perceived recovery trajectories following URS and PCNL. No clear differences in overall recovery duration were demonstrated between procedures within the limitations of a small and heterogeneous sample. PCNL may be associated with a longer recovery in selected domains; however, larger studies are required to confirm these findings. The questionnaire requires further validation before clinical implementation.

## 1. Introduction

Urolithiasis or kidney stone disease is a highly prevalent disease worldwide [1,2].

When surgical intervention is required, retrograde ureterorenoscopy (URS) and percutaneous nephrolithotomy (PCNL) are commonly employed treatment modalities. Both procedures have been extensively compared with respect to stone-free rates, complications, and costs [3,4,5]. Although urolithiasis and its treatment significantly affect quality of life, most available evidence on postoperative recovery after endourological stone surgery relies on indirect measures such as hospital stay, analgesic use, or sick leave, often derived from retrospective or heterogeneous cohorts. As a result, detailed prospective data describing symptom-based recovery trajectories and temporal patterns of postoperative complaints remain limited [6]. In addition, existing quality-of-life studies in this population are often procedure-specific or do not capture the duration and temporal course of postoperative symptoms in sufficient detail [7,8].

Several validated patient-reported outcome measures (PROMs) are available for patients with kidney stone disease, including the Wisconsin Stone Quality of Life (WISQOL) questionnaire and generic PROMIS instruments [9,10]. These instruments primarily assess health-related quality of life and symptom burden, but are not designed to capture short-term postoperative recovery trajectories or specific recovery milestones following endourological procedures, such as return to work, resumption of daily activities, pain resolution, fatigue, and overall perceived recovery [6,11,12].

Therefore, an exploratory procedure-specific questionnaire was developed to evaluate postoperative recovery patterns following URS and PCNL and to identify clinically relevant domains of recovery. The results of this study may contribute to further refinement and future validation of this instrument.

In addition, previous studies have reported indirect measures of recovery after endourological stone surgery, including return-to-work time, analgesic use, and hospital stay. Reported sick leave typically ranges from approximately 5–7 days after ureterorenoscopy, whereas percutaneous nephrolithotomy is associated with longer convalescence periods of approximately 10–14 days or more [4,13]. However, these endpoints are mainly derived from administrative or retrospective data and do not capture symptom resolution, recovery trajectories, or day-to-day patient-perceived recovery in a structured manner.

The aim of this study was to prospectively evaluate recovery after URS and PCNL in routine clinical practice, in order to provide the next step in more realistic expectations for patients and to support clinicians in preoperative counseling.

## 2. Methods

### 2.1. Study Design

This prospective longitudinal questionnaire study was conducted at the urology departments of three hospitals in the Netherlands: an academic referral center for complex stone disease (UMCG), a large teaching hospital (MZH), and a regional hospital (WZA). The study was approved by the local medical ethics committee of the UMCG, which determined that no extensive ethical review was required.

This study was designed as an exploratory pilot study aimed at characterizing postoperative recovery following URS and PCNL and evaluating the feasibility of collecting patient-reported recovery data using a longitudinal, questionnaire-based approach. No formal sample size or power calculation was performed prior to study initiation.

### 2.2. Participants, Inclusion, and Recruitment

Between July 2020 and September 2021, patients scheduled for URS or PCNL for renal stones were screened for inclusion. Eligibility criteria were age ≥ 18 years, treatment for kidney stones, ability to read and speak Dutch (or supported by a mentor/caregiver), and mental competence to complete questionnaires. Patients treated for ureteral stones were excluded.

Patients were enrolled during hospital admission after surgery. Only the first treatment procedure was included for patients undergoing multiple procedures during the study period.

### 2.3. Data Collection

Data were collected using REDCap (Research Electronic Data Capture; Vanderbilt University Medical Center, Nashville, TN, USA; available at https://projectredcap.org; accessed on 6 July 2026) a secure web-based research data platform. Questionnaires were distributed electronically two weeks after surgery, with automated reminders for non-response. Patients without email access received postal questionnaires. If postoperative complaints persisted at two weeks, follow-up telephone interviews were conducted at five and, if needed, eight weeks postoperatively.

Demographic, clinical, and stone characteristics were extracted from electronic medical records.

### 2.4. Endpoints

The primary endpoints of this study were time to full recovery, duration of postoperative pain and duration of fatigue. Secondary outcomes included time to return to work and time to resume sports activities. Full recovery was defined as the patient-reported complete absence of all postoperative complaints and a return to their preoperative baseline state. This was assessed using a direct questionnaire item asking patients whether they considered themselves fully recovered.

Duration of pain and fatigue was assessed using structured patient-reported questions in which patients indicated the number of days after surgery during which they experienced pain or fatigue above their individual preoperative baseline level. Responses were collected using predefined categorical time intervals to standardize reporting across participants.

All outcomes were therefore based on patient-reported perceptions of recovery relative to baseline and reflect subjective recovery experiences rather than objective clinical measurements.

### 2.5. Questionnaire

A 62-item questionnaire was developed for this study as an exploratory instrument to evaluate postoperative recovery following ureterorenoscopy (URS) and percutaneous nephrolithotomy (PCNL). The questionnaire was designed to capture both general and procedure-specific aspects of recovery. The instrument was partly based on the SF-36 [14] but was specifically designed as an exploratory recovery assessment tool, tailored to the study’s research question. At the time of study design, no validated instrument was available that combined postoperative symptom burden with time-specific recovery milestones following URS and PCNL. Therefore, this study aimed to provide preliminary data to inform future refinement and formal validation of a recovery-specific questionnaire.

Items were derived from domains included in established quality-of-life instruments, including physical functioning, fatigue, emotional well-being, and sleep quality. These were supplemented with procedure-specific questions addressing postoperative urological symptoms such as flank pain, dysuria, hematuria, and recovery milestones, including return to work, return to sports activities, and perceived overall recovery.

Symptoms were defined as self-reported complaints relative to the patient’s usual preoperative baseline state.

Although based on established quality-of-life domains, the questionnaire was not formally validated prior to use. No pilot testing, cognitive interviewing, or Delphi consensus procedure was performed. The instrument was intentionally implemented as an exploratory tool to generate hypothesis-forming data on recovery trajectories after endourological stone surgery. An example of one of the questions is: How many days after the surgery did you have more pain than normal (0 to 14 days or >14 days)? The definition of ‘Normal’ was based on patients’ judgment and responses focused on postoperative complaints only; preoperative symptoms were explicitly excluded.

### 2.6. Statistics

All statistical analyses were performed using the Statistical Package for the Social Sciences for Windows (IBM SPSS Statistics for Windows, version 24.0; IBM Corp., Armonk, NY, USA). Continuous variables were analyzed using the Mann–Whitney U test or independent-samples *t*-test, depending on distribution. Categorical variables were analyzed using the chi-square test. Normality was assessed using probability density functions and graphical methods. A two-sided alpha level of 0.05 was used.

### 2.7. Surgery and Follow-Up

Patients received standard perioperative care according to institutional protocols and national guidelines. Surgical techniques and equipment differed slightly between centers. At MZH (Groningen, The Netherlands), a thulium fiber laser (SOLTIVE™ SuperPulsed Laser System; Olympus Corporation, Hachioji, Tokyo, Japan) was used, whereas low-energy Holmium: YAG lasers (Swiss LaserClast^®^; EMS Electro Medical Systems SA, Nyon, Switzerland) were employed at UMCG (Groningen, The Netherlands) and WZA (Assen, The Netherlands).

The choice of URS or PCNL was made according to routine clinical practice. Besides stone size, factors such as stone location, anatomical abnormalities, previous unsuccessful stone treatments, and surgeon preference were taken into account when selecting the surgical approach. For PCNL, an outer tract diameter of 24 Fr was used at MZH and WZA. At UMCG, mini-PCNL (outer diameter 16.5 Fr) and ultra-mini PCNL (13.5 Fr) were also performed. For analysis purposes, mini-PCNL and ultra-mini PCNL were combined, as only one ultra-mini PCNL case was included.

Only patients with renal stones were included; all URS procedures were performed using a flexible ureteroscope. Postoperative imaging was obtained according to clinical indication and was not mandated by the study protocol.

## 3. Results

### 3.1. Cohort Characteristics and Responses

Of 234 consenting patients, 131 (56%) completed the questionnaire. Seventy-one patients with renal stones were included in the analysis: 34 after URS and 37 after PCNL.

The mean age of the cohort was 57.5 years (SD 13.4), and 37 patients were male (52.1%). The mean body mass index (BMI) was 27.2 kg/m^2^ (SD 4.8). Detailed baseline characteristics and comparisons between the URS and PCNL groups are presented in Table 1.

Pain was the primary indication for surgical treatment in 34 patients (47.9%), while infection was the indication in seven patients (9.9%). Thirty patients were asymptomatic and underwent preventive surgical treatment. Although 28 patients (39.4%) had bilateral renal stones, all patients were treated in a single renal unit. Forty-two patients (59.2%) had more than one stone in the treated kidney, and 52.9% of stones measured ≥9 mm.

During follow-up, postoperative imaging was performed using CT scans in 52 patients and ultrasonography in 3 patients; the remaining 16 patients did not undergo postoperative imaging. A total of nine postoperative complications were recorded: seven urinary tract infections, one case of severe bleeding, and one ureteric perforation.

### 3.2. Recovery

An overview of recovery outcomes for the entire cohort is presented in Table 2. Below, recovery outcomes are described separately for the URS and PCNL groups, including comparisons between procedures.

Baseline patient characteristics did not differ significantly between the URS and PCNL groups with respect to sex distribution, age, body mass index (BMI), or ASA score. No significant differences were observed in stone number, stone size, or the proportion of patients with paid employment or participation in sports activities.

General anesthesia was used more often in the PCNL group than in the URS group (100% vs. 76.4%). Among patients who received general anesthesia, the duration exceeded 60 min more frequently in the PCNL group than in the URS group (94.4% vs. 46.2%). Infection was more often the indication for treatment in the PCNL group compared with the URS group.

No statistically significant differences were observed between groups with respect to postoperative complication rates, the proportion of patients receiving a JJ catheter, or ureteral catheter placement. Stone-free rates after surgery also did not differ significantly between groups; however, follow-up imaging strategies were not uniform across all patients. Stone-free status was defined as the absence of any visible residual calculi on follow-up imaging; however, not all patients underwent the same imaging modality during follow-up.

Among the 37 patients who underwent PCNL, the median time to resolution of all postoperative complaints was 15 days. Among the 34 patients treated with URS, the corresponding median duration was 13 days; this difference was not statistically significant. Median duration of postoperative pain was 4 days after PCNL and 2 days after URS. Median duration of fatigue was 12 days in the PCNL group and 7 days in the URS group.

The median duration of micturition complaints was 0 days after PCNL and 3 days after URS. Patients reported being housebound for a median of 8 days following PCNL and 4.5 days following URS.

In the PCNL group, 16 patients had paid employment, with a median time to full return to work of 13 days. In the URS group, 19 patients had paid employment, and the median time to full return to work was 9 days. Median time to resumption of sports activities was 17 days after PCNL (among 26 patients) and 10 days after URS (among 25 patients).

Overall, the number of days to full recovery did not differ significantly between the PCNL and URS groups. A significant difference was observed in the duration of absence from work, which was shorter in the URS group (*p* = 0.014); however, no significant difference was found in the time to full return to work.

## 4. Discussion

This prospective multicenter study provides one of the first prospective insights into patient-perceived recovery trajectories following URS and PCNL. More than 95% of patients reported postoperative complaints, with fatigue being the most frequently reported symptom. The median time to full recovery was approximately two weeks, highlighting the substantial short-term impact of these commonly performed urological procedures on patients’ daily functioning.

The observation that no statistically significant difference in overall recovery time was observed between URS and PCNL should be interpreted cautiously. Importantly, this study was not designed or powered as an equivalence study, and the absence of statistically significant differences must therefore not be interpreted as evidence of equivalence between procedures. Rather, these findings indicate that within the constraints of the present sample size and study design, differences in recovery duration could not be statistically demonstrated.

Previous research has mainly focused on quality-of-life outcomes or absence from work following urolithiasis treatment. Jacobs et al. used a standardized quality-of-life questionnaire and reported that 61.1% of patients experienced difficulties performing usual daily activities after URS. During the first two postoperative weeks, 70.6% of patients used pain medication, and patients missed an average of 5.61 working days following surgery [13]. The authors concluded that urolithiasis treatment has a considerable impact on patients’ lives, particularly regarding usual activities and pain or discomfort, and emphasized the need for further research. The present study contributes additional, complementary data by focusing more specifically on the duration and course of postoperative recovery symptoms.

All primary outcomes were patient-reported and inherently subjective in nature, as they reflect patients’ perceived recovery rather than objective clinical measurements. However, this approach is consistent with contemporary patient-reported outcome research, which emphasizes the importance of capturing subjective recovery experiences.

Kannopka et al. primarily investigated the costs associated with different urolithiasis treatments and reported urolithiasis-related sick leave following extracorporeal shockwave lithotripsy (SWL), URS and PCNL. They observed significantly more sick-leave days after PCNL (13.0 days) compared with URS (6.8 days), results that are in line with the findings of the current study [4].

Wilhelm et al. reported a longer hospital stay following ultra-mini PCNL compared with URS, while postoperative pain did not differ significantly between groups—findings comparable to our results for conventional PCNL [15]. In this study, a subgroup comparison between conventional PCNL and mini- or ultra-mini PCNL was not conducted due to the limited number of patients in these groups.

To enhance representativeness, patients were recruited from an academic referral center for complex stone disease, a large teaching hospital, and a regional hospital. Together, these institutions account for a substantial proportion of urolithiasis-related urological care in the Netherlands. Nevertheless, it remains uncertain whether the study population fully represents all patients undergoing stone surgery in routine clinical practice.

Several limitations should be considered when interpreting these findings. Although no statistically significant differences were found in baseline patient or stone characteristics between the URS and PCNL groups, procedural characteristics and indications for surgery differed substantially. In particular, differences in treatment indication, anesthesia type, and operative duration may have influenced postoperative recovery. Given the exploratory pilot design and the relatively small sample size, multivariable adjustment for these potential confounders was not considered sufficiently robust. Consequently, residual confounding cannot be excluded, and comparisons between URS and PCNL should be interpreted with caution. In addition, despite clear instructions to report only postoperative complaints, it cannot be excluded that some patients may have inadvertently included symptoms related to their stone disease prior to surgery, such as pain, potentially leading to overestimation of surgery-related complaints.

Stone size categories above 9 mm were combined for statistical analysis due to limited subgroup sizes, which may have introduced heterogeneity within this group; however, no stones exceeded 25 mm in this cohort.

Notably, postoperative imaging was not standardized as part of the study protocol, as patients received routine clinical follow-up according to local practice. Consequently, follow-up imaging data were unavailable for a proportion of patients. Residual stone fragments may contribute to ongoing symptoms, discomfort, or delayed recovery and could therefore have influenced patient-reported outcomes. As stone-free status was defined as the complete absence of residual stone fragments, the impact of residual fragments on postoperative recovery could not be fully assessed in all patients and should be considered when interpreting the results.

Furthermore, this exploratory pilot study was conducted without a formal sample size calculation, and the relatively small sample size may have limited the ability to detect subtle differences between treatment groups. In addition, the response rate was 56%, introducing the possibility of selection bias. As non-responders did not provide informed consent, their characteristics could not be evaluated. Therefore, the generalizability of the findings should be interpreted with caution. Finally, a non-validated questionnaire was used in this study. This instrument was intentionally developed as an exploratory outcome measure, tailored to address recovery-specific questions that were not adequately covered by existing validated questionnaires at the time of study design. While this approach allowed an assessment of different dimensions of patient-reported recovery, as outlined in the Introduction, there is currently a lack of validated instruments specifically capturing short-term, procedure-specific recovery trajectories following endourological stone surgery. The present questionnaire was therefore developed to address this gap and to provide preliminary data for future instrument refinement and validation. It should therefore be regarded as a hypothesis-generating tool intended to identify relevant domains of postoperative recovery rather than as a validated patient-reported outcome measure.

While this approach allowed an assessment of different dimensions of patient-reported recovery, the exploratory nature of the study and the absence of formal validation should be considered when interpreting the findings. Future validation of this questionnaire is warranted and represents an important next step to strengthen its applicability in both research and clinical practice. In addition, the findings of the present study may be used to further refine the questionnaire and identify the most clinically relevant recovery domains for future validation studies. The present questionnaire should therefore be regarded as a hypothesis-generating instrument intended to identify relevant domains of postoperative recovery rather than as a validated patient-reported outcome measure.

## 5. Conclusions

Despite its limitations, this study provides one of the first prospective insights into patient-perceived recovery trajectories following URS and PCNL and contributes to the currently limited literature on postoperative recovery after stone surgery.

PCNL is generally considered a more invasive procedure than URS and is associated with higher complication rates and higher stone-free rates [16,17,18]. In the present study, however, within the limitations of this exploratory study, differences in overall recovery duration between URS and PCNL were not clearly demonstrated, although larger studies are needed to confirm these observations.

A trend toward longer inability to resume sports activities was observed in the PCNL group (*p* = 0.061). In addition, PCNL was associated with a longer hospital stay, a longer duration of fatigue, and a longer absence from work compared with URS.

Selected postoperative symptoms showed no significant differences; however, larger cohorts are needed to further clarify recovery patterns. The present findings should therefore be considered hypothesis-generating rather than definitive. Validation and refinement of the questionnaire will be important next steps to better characterize postoperative recovery and identify patients at risk for prolonged recovery [19], supporting more patient-oriented counseling and procedure selection.

## Figures and Tables

**Table 1:** Population and surgery characteristics.

Population Characteristics		Total	PCNL	URS	*p*-Value *
*n*		71	37	34	
Male; *n* (%)		37 (52.1%)	19 (51.4%)	18 (52.9%)	0.89
Mean age years (SD)		57.5 (13.4)	55.3 (15.4)	59.6 (13.1)	0.22
Average BMI (kg/m^2^) (SD)		27.2 (4.8)	27.2 (5.5)	27.2 (3.95)	1.00
ASA-class ^^^	ASA-1 *n* (%)	13 (18.3%)	5 (13.5%)	8 (23.5%)	0.41
	ASA-2 *n* (%)	37 (52.1%)	19 (51.4%)	18 (52.9%)	
	ASA-3 *n* (%)	21 (29.6%)	13 (35.1%)	8 (23.5%)	
Paid employment; *n* (%)		35 (49.3%)	16 (43.2%)	19 (55.9%)	0.39
Sports (including walking); *n* (%)		51 (77.3%)	26 (72.2%)	25 (83.3%)	0.28
Surgery characteristics		Total	PCNL	URS	*p*-value *
Number of stones (treated kidney)	1	29 (40.8%)	12 (32.4%)	17 (50%)	0.13
	>1	42 (59.2%)	25 (67.6%)	17 (50%)	
Stone size	1–4 mm; *n* (%)	5 (7.1%) (missing *n* = 1)	1 (2.8%) (missing *n* = 1)	4 (11.8%) (missing *n* = 0)	0.12
	5–9 mm; *n* (%)	28 (40.0%) (missing *n* = 1)	12 (33.3%) (missing *n* = 1)	16 (47.1%) (missing *n* = 0)	
	>9 mm; *n* (%)	37 (52.9%) (missing *n* = 1)	23 (63.9%) (missing *n* = 1)	14 (41.2%) (missing *n* = 0)	
Treatment indication	Pain	34 (47.9%)	16 (43.2%)	18 (52.9%)	**0.02**
	Infection	7 (9.9%)	7 (18.9%)	0 (0%)	
	Prevention/other	30 (42.3%)	14 (37.8%)	16 (47.1%)	
Anesthesia (general/spinal)		63/8	37/0	26/8	**0.002**
Duration of general anesthesia	≤60 min	16 (25.8%) (missing *n* = 1)	2 (5.6%) (missing *n* = 1)	14 (53.8%) (missing *n* = 0)	**<0.001**
	>60 min	46 (74.2%) (missing *n* = 1)	34 (94.4%) (missing *n* = 1)	12 (46.2%) (missing *n* = 0)	
Exit strategy after operation	JJ-stent	44 (62.0%)	21 (56.8%)	23 (67.6%)	0.35
	CAD	43 (60.6%)	21 (56.8%)	22 (64.7%)	0.50
	Ureteral catheter	24 (33.8%)	12 (32.4%)	12 (35.3%)	0.80
	Nephrostomy tube	7 (2 chronic) (9.9%)	7 (2 chronic) (18.9%)	0 (0%)	**0.012**
Days of hospitalization	0 or 1 night	52 (73.2%)	21 (56.8%)	31 (91.2%)	**0.001**
	2 nights or more	19 (26.8%)	16 (43.2%)	3 (8.8%)	
Complications; *n* (%)		9 (12.7%)	7 (18.9%)	2 (5.9%)	0.16
Stone-free upon follow-up imaging *n* = 56 ^#^		31 (43.6%) (missing *n* = 15)	17 (45.9%) (missing *n* = 9)	14 (41.1%) (missing *n* = 6)	0.63

* *p* value < 0.05 = significant difference between URS and PCNL. Statistically significant results are shown in bold in the table above. ^^^ ASA= ASA Physical Status Classification System of the ‘American Society of Anesthesiologists’. ^#^ missing data: Follow-up imaging was not available for all participants.

**Table 2:** Recovery durations.

Recovery Duration in Days	Total Median (*n* = 71)	PCNL Median (*n* = 37)	URS Median (*n* = 34)	*p*-Value *
Full recovery	15	15	13	0.27
	9–29	10–30	7–21.25	
	(missing: 0)	(missing: 0)	(missing: 0)	
Pain	3	4	2	0.36
	0–11	0–12	0–8.25	
	(missing: 0)	(Missing: 0)	(missing: 0)	
Fatigued	9	12	7	**0.016**
	3–15	8–19.5	2.75–13	
	(missing: 0)	(missing: 0)	(missing: 0)	
Trouble sleeping	4	4	4	0.89
	0–10	0–11.5	0–8.25	
	(missing: 0)	(missing: 0)	(missing: 0)	
Micturition complaints	2	0	3	0.36
	0–8	0–8	0–7.25	
	(missing: 0)	(missing: 0)	(missing: 0)	
Worsened mood	0	0	0	0.90
	0–4	0–6.5	0–4	
	(missing: 0)	(Missing: 0)	(missing: 0)	
More housebound	7	8	4.5	0.10
	0–10	1–13.5	0–8.5	
	(missing: 0)	(missing: 0)	(missing: 0)	
Hobbies completely possible	7	7	7.5	0.94
	3–13	3–13	2.25–12.75	
	(missing: 28) ^#^	(missing: 14) ^#^	(missing: 14) ^#^	
Sports completely possible	14	17	10	0.06
	8.22	13–30	7–19.25	
	(missing: 32) ^#^	(missing: 18) ^#^	(missing: 14) ^#^	
Unable to work	10	12.5	7	**0.014**
	5.5–13	7–15	3–11	
	(missing: 34) ^#^	(missing: 17) ^#^	(missing: 17) ^#^	
Full return work	11.5	13	9	0.10
	6.75–15	8.5–15	5.75–13	
	(missing: 33) ^#^	(missing: 17) ^#^	(missing: 16) ^#^	

All data are presented as median (Interquartile range). ^#^ Missing data had multiple causes, including non-response to researcher contact and the absence of work or sports activities among several participants prior to surgery. * *p* value < 0.05 = significant difference. Statistically significant results are shown in bold in the table above.

## Data Availability

The original contributions presented in this study are included in the article. Further inquiries can be directed to the corresponding authors.
